# Imaging Findings of Duodenal Duplication Cyst Complicated with Duodenal Intussusception and Biliary Dilatation

**DOI:** 10.1155/2016/3069576

**Published:** 2016-02-17

**Authors:** Eduardo Torres Diez, Raúl Pellón Dabén, Juan Crespo Del Pozo, Francisco José González Sánchez

**Affiliations:** ^1^Department of Radiology, Central de Asturias University Hospital, Oviedo, Spain; ^2^Department of Radiology, Marqués de Valdecilla University Hospital, Santander, Spain

## Abstract

Duodenal duplication cyst is an extremely rare congenital anomaly usually diagnosed in childhood. However, it may remain asymptomatic for a long period. In adults it usually manifests with symptoms related to complications as pancreatitis, jaundice, or intussusception. We present the radiology findings of a patient with a duodenal intussusception secondary to a duplication cyst. The usefulness of the magnetic resonance (MR) in this case is highlighted.

## 1. Case Report

A 33-year-old woman with a history of intermittent jaundice was referred to our radiology department. She had complained of recurrent abdominal pain for 5 years. At the age of 30 years she had been diagnosed of cholelitiasis by an ultrasound performed in another centre. Her liver function tests were abnormal with slight elevation of bilirubin.

**Figure 1:**
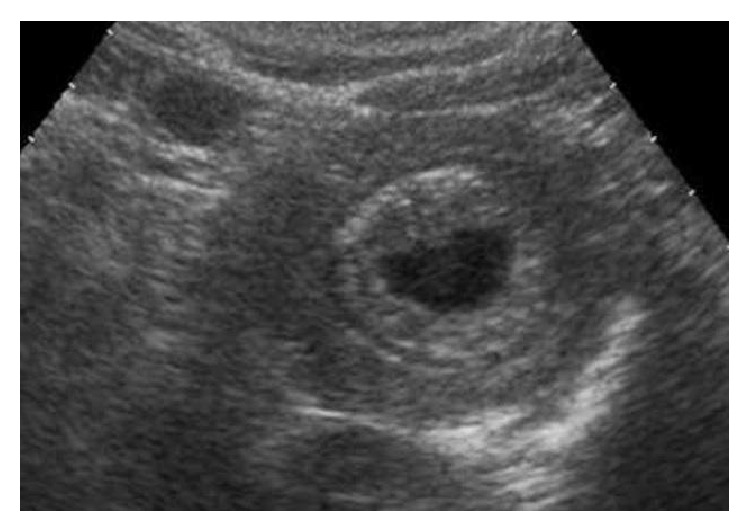
Abdominal ultrasound image depicts the typical image of intussusception with a simple cyst inside.

First an abdominal ultrasound was realized. It showed an intussusception involving the duodenum and proximal jejunum. An anechoic cystic lesion was observed inside the lesion (Figure 1). Besides, the biliary tree was dilated.

**Figure 2:**
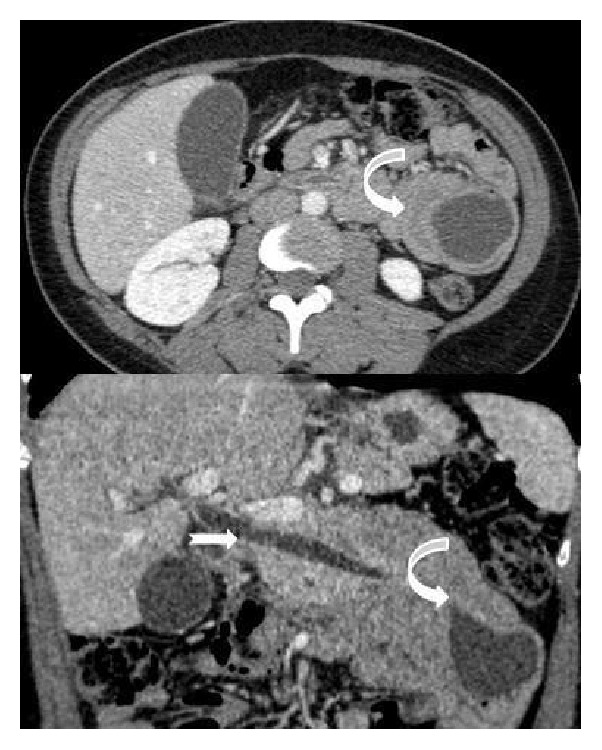
Axial and coronal CT shows that the distal duodenum with surrounding fat, vessels, and the pancreatic head were invaginated into the proximal jejunum (curve arrow). The common bile duct is dilated (single arrow).

**Figure 3:**
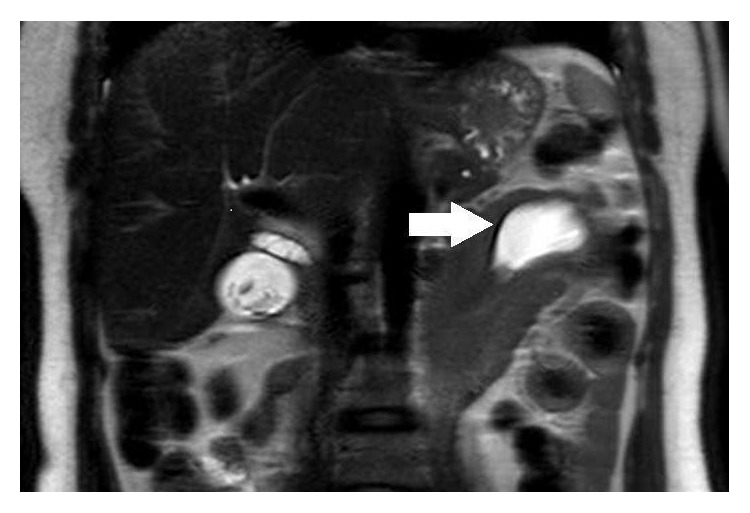
Coronal T2 weighted single-shot turbo spin echo RM image confirms the cyst (arrow) as the cause of intussusception and biliary dilatation.

**Figure 4:**
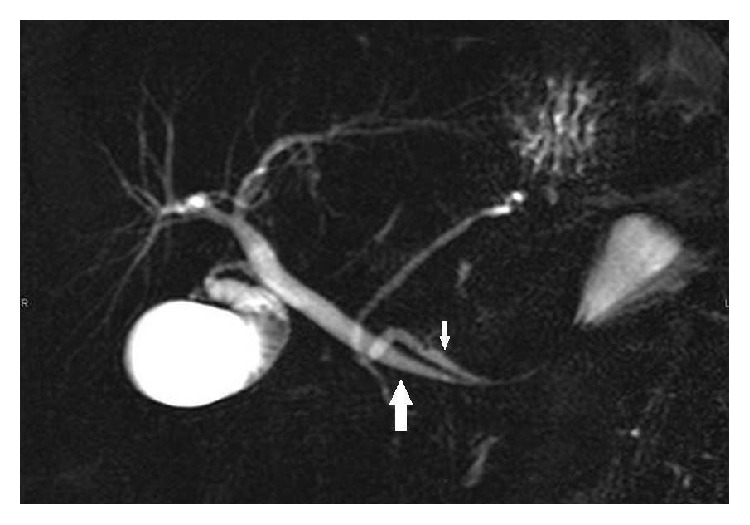
MR Cholangiopancreatography reveals that the common bile duct (large arrow) and the main pancreatic duct (small arrow) are pulled down and leftward into the elongated duodenum.

**Figure 5:**
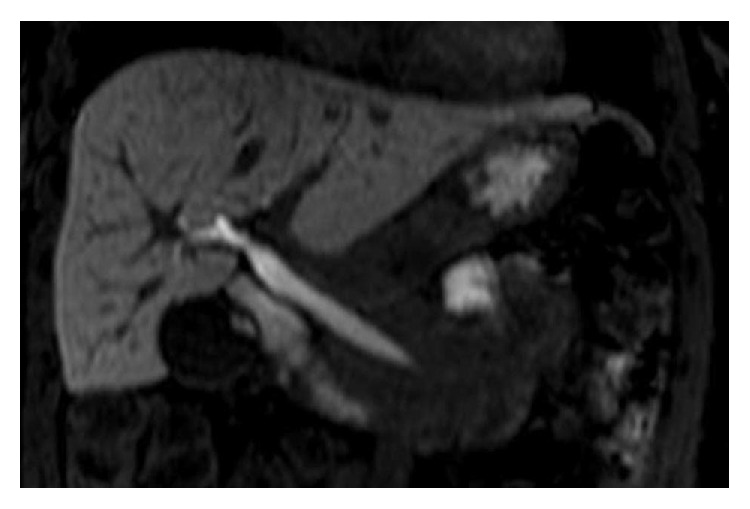
Coronal multiplanar reformation Gd-EOB-DTPA enhanced T1 weighted 3D GRE image obtained 30 minutes after injection shows contrast material in bile duct and duodenal lumen. Not inside the cyst.

**Figure 6:**
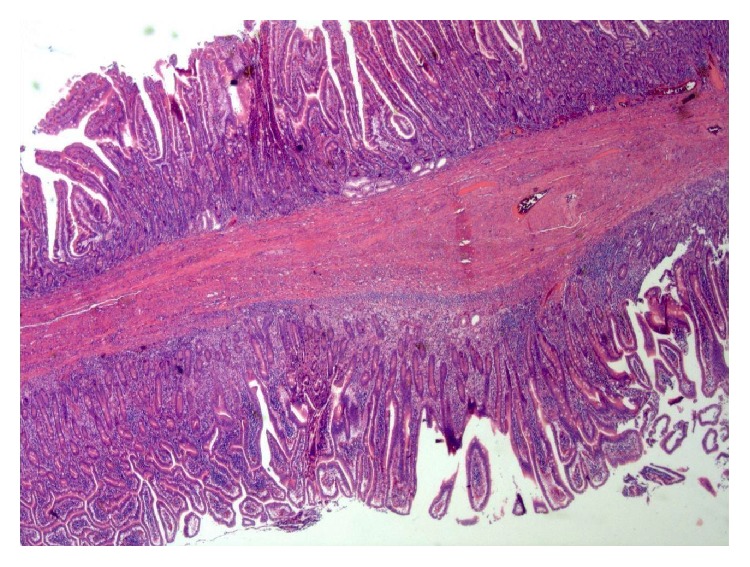
An image of the histological examination shows characteristic findings of a duplication cyst and a double muscle layer, with their mucosal and submucosal layers.

The study was continued with computed tomography with contrast and obtainment in portal phase, and after a complementary magnetic resonance (MR) with axial and coronal T2 weighted single-shot turbo spin echo, MR Cholangiopancreatography and contrast enhancement sequences were performed. Gadolinium ethoxybenzyl diethylenetriamine pentaacetic acid was used for the dynamic study. We realized vascular acquisitions and a delayed hepatobiliary phase. The axial CT images and T2 weighted sequences confirmed that the distal duodenum with surrounding fat, vessels, and the pancreatic head were invaginated into the proximal jejunum. Inside the intussusception a cystic lesion was also visualized. The stomach was not distended (Figures 2 and 3). The MR Cholangiopancreatography depicted that the extrahepatic bile duct was slightly dilated and pulled leftward towards the cyst mass. The contrast enhancement sequences corroborated the simple cystic nature of the cause of intussusception (Figure 4). In the hepatocytic acquisition phase the contrast filled the biliary tree and the bowel lumen, but not inside the cyst lumen (Figure 5). An open surgery was the treatment elected. A laparotomy was realized and a cystic lesion measuring 4 × 4 × 3 cm was removed. At histopathologic analysis the cyst presented definitive criteria of duplication cyst (Figure 6). The postoperative period was uneventful and ten days later the patient was discharged.

## 2. Discussion

Duplication cyst is a rare congenital condition that forms during the embryonic period of alimentary tract development. Most cysts are 2 to 4 cm in size. They occur frequently in the distal ileum. Conversely duodenal duplication cysts are very uncommon and represent only 2 to 12% of all digestive tract duplications [1, 2]. Most of the duodenal duplication cyst are located on the second or third part of duodenum and share muscle layers. They are commonly cystic and usually communicate with pancreatic or bile ducts. The presence of a communication with duodenal lumen is not frequent [1, 2].

Duodenal duplication cysts are usually diagnosed in the childhood. However they may remain asymptomatic until adulthood. Clinical manifestations in adults of duodenal duplication cyst are usually nonspecific and may easily be misinterpreted. Abdominal pain, nausea, and vomiting are the most common symptoms. They can present with a complication, pancreatitis and cholestasis being the most frequent. Intussusception, gastrointestinal bleeding, and cyst infection are also common [1]. Symptoms are usually of long duration even if the intussusception is present [3]. Imaging techniques usually suggest the diagnosis, but it must be confirmed at histopathologic analysis. The criteria required for the definitive diagnosis are the presence of alimentary mucosal lining, a smooth muscle coat, and an intimate attachment to the native gastrointestinal tract [4].

Intussusception in adults is rare and there is an underlying disorder in 90% of cases [3]. A mass can lead to the telescoping of one bowel segment over the adjacent. Duodenojejunal intussusceptions are rarely encountered because of fixation of a large portion of the duodenum to the retroperitoneum [3, 5–7]. Duodenojejunal intussusceptions are almost always triggered by a benign cause as lipoma, adenoma, or hamartoma. In our case an enteric duplication cyst acts as the lead point. However, malignant causes have also been described [3, 5, 6].

Imaging studies are essential for a preoperative diagnosis. An ultrasonography is usually the first imaging technique performed. The ultrasound depicts the pathognomonic bowel within the bowel of the intussusceptions and can show the cystic nature of the duplication cyst. The localization of intussusception usually changes during examination. Besides it may be possible to see peristalsis in the wall caused by the muscular layer [8]. Seldom, duplication cyst may present as an echogenic mass [9]. The CT and MRI can confirm the presence of intussusception and with contrast enhance the cystic nature of duplication cyst. They have a better spatial resolution than ultrasound and detect the presence of complications like pancreatitis, being very useful for preoperative planning. The MR Cholangiopancreatography provides additional information through better resolution of biliary tree. The addition of the hepatocyte-specific contrast allows the visualization of contrast inside the cyst which narrows the differential diagnosis. Only choledochal cyst which is in communication with biliary tree can present contrast filling. Nonetheless, it has not been described as a cause of intussusception. Other possible and more frequent cystic lesions in epigastric area like pseudocyst, mesenteric cyst, or pancreatic cystic tumours can be completely excluded.

A treatment is necessary when adults present with symptomatic intussusception. Open surgical intervention is the most common technique for duplication duodenal cyst. Complete excision is usually frequent. This is the treatment of choice in case of complicated cyst intussusception [2]. Other treatment options include surgical marsupialisation, surgical resection, pancreaticoduodenectomies, and endoscopic marsupialisation [1].
